# High-Resolution Mass Spectrometry-Based Proteomic Profiling of *Lemna minor*: Insights into Protein Composition, Extraction Strategies and Allergenicity Risk

**DOI:** 10.3390/foods15213812

**Published:** 2026-10-24

**Authors:** Antonella Lamonaca, Elisabetta De Angelis, Massimiliano D’Imperio, Giuseppe Di Cuia, Francesco Serio, Rosa Pilolli

**Affiliations:** 1 Institute of Sciences of Food Production, National Research Council of Italy, Via Amendola 122/O, 70126 Bari, Italy; antonellalamonaca@cnr.it (A.L.); massimiliano.dimperio@cnr.it (M.D.); g.dicuia1@phd.uniba.it (G.D.C.); francesco.serio@cnr.it (F.S.); rosa.pilolli@cnr.it (R.P.); 2 Department of Soil, Plant, and Food Sciences, University of Bari Aldo Moro, Via G. Amendola 165/a, 70126 Bari, Italy

**Keywords:** alternative proteins, duckweed, *Lemna minor*, proteomics, protein extraction, in silico allergenicity

## Abstract

*Lemna minor* L. (LM) has recently gained considerable interest as a sustainable protein-rich novel food; however, information on its proteome remains limited due to the scarcity of species-specific sequence databases and the lack of comprehensive mass spectrometry-based investigations. This study provides the first extensive proteomic characterization of LM using high-resolution tandem mass spectrometry and evaluates the influence of different extraction strategies on proteome coverage. LM was cultivated under controlled conditions and harvested after 10 days, yielding 1.00 ± 0.15 kg/m^2^ and a protein content of 35% on a dry weight basis. To maximize proteome coverage, proteins were extracted at different pH values (7, 9, and 11) by ultrasound-assisted extraction and by thermostatic extraction under shaking. Although all protocols showed comparable protein contents (8.2–9.1 mg/mL) and extraction efficiencies (58.6–65.1%), hierarchical clustering, Gene Ontology analysis, SDS-PAGE profiling, and in-gel protein identification revealed differences in the recovery of specific protein classes, supporting the integration of data from multiple extraction approaches. Bottom-up shotgun proteomics identified 1211 proteins and 4149 validated peptides, representing the most comprehensive LM proteome dataset reported to date. Most proteins were associated with photosynthesis, carbon fixation, energy metabolism, cellular structure, and stress-response pathways. An in silico allergenicity assessment identified 40 proteins that were homologous to known food allergens, with chlorophyll a/b-binding protein showing the highest abundance in peak signal and similarity to the celery allergen Api g 3. Overall, this work provides a valuable resource for future nutritional, functional, and safety evaluations of duckweed-derived foods.

## 1. Introduction

The global population is expected to reach 9.8 billion by 2050, increasing the demand for safe, nutritious, and sustainable food systems [1]. Proteins are of particular concern, as the current requirements are predominantly met through animal-derived foods whose production contributes substantially to greenhouse gas emissions and water consumption [2]. Consequently, growing environmental and health awareness has accelerated interest in alternative protein sources, with plant proteins emerging as promising candidates for sustainable food formulations [3].

Among these, duckweeds (family *Lemnaceae*) have attracted increasing attention due to their rapid growth, high biomass productivity, and elevated protein content. This family comprises 37 species distributed among five genera (*Spirodela*, *Landoltia*, *Lemna*, *Wolffiella*, and *Wolffia*) [4]. Under favorable conditions, duckweeds can double their biomass within a few days, surpassing the growth rates of most terrestrial crops [5]. Furthermore, several species exhibit protein contents exceeding 40% dry weight and possess amino acid profiles that closely match WHO recommendations, making them attractive alternatives to conventional plant and animal protein sources [6,7,8].

Beyond their nutritional value, duckweeds offer important environmental advantages, including efficient water use, suitability for controlled cultivation systems, and high protein yields per cultivation area [7,9]. These characteristics have stimulated growing regulatory and industrial interest in Europe. Following extensive safety evaluations by the European Food Safety Authority (EFSA), *Wolffia globosa* and *Wolffia arrhiza* were recognized as traditional foods from third countries in 2021 [10], while protein concentrates from *Lemna gibba* and *Lemna minor* were authorized as novel foods in 2023 [11]. Subsequently, whole *L. gibba* and *L. minor* were included in the EU Novel Food list after EFSA concluded that safety concerns associated with manganese content could be adequately managed [12].

Among duckweed species, *Lemna minor* (LM) is one of the most widespread and adaptable. Its rapid growth, broad tolerance to environmental conditions, and ability to modulate biochemical composition according to cultivation parameters make it particularly attractive for food applications [4,13,14]. Importantly, potential safety concerns associated with heavy metals, contaminants, and antinutritional factors can be significantly mitigated through controlled cultivation and appropriate processing technologies [7,15].

The recent authorization of LM-derived ingredients has stimulated research on protein extraction and recovery, with reported yields ranging from 16% to 45% depending on cultivation and processing conditions [7,16,17]. Nevertheless, knowledge of the LM proteome remains limited. Current information is largely restricted to electrophoretic studies and the characterization of major proteins such as RuBisCO and chlorophyll-binding proteins [5], while other proteomic investigations have focused on ecotoxicological stress responses or have explored omics approaches in related Lemnaceae species [18,19]. Moreover, no reference proteome is currently available for LM, and only a limited number of fully sequenced protein entries have been deposited in public databases. Although EFSA has considered the species to present a low allergenic risk based on available evidence [20], comprehensive proteome characterization and allergenicity assessment remain necessary.

Therefore, the aim of this study was to provide a comprehensive proteomic characterization of LM whole biomass using high-resolution mass spectrometry and advanced bioinformatic workflows. In parallel, the allergenic potential of the identified proteome was assessed through an in silico approach based on the latest EFSA guidelines [21]. To maximize proteome coverage, different sample preparation strategies were tested and combined with complementary shotgun and gel-based proteomic approaches.

## 2. Materials and Methods

### 2.1. Lemna minor Growth Conditions

*Lemna minor* (LM) growth was conducted in 2025 in a walk-in growth chamber at the Institute of Sciences of Food Production (ISPA-CNR) in Bari (BA, Italy), with internal dimensions of 260 × 284 × 253 cm (length, width, and height, respectively). Duckweed was grown in plastic trays (1728 cm^2^ each), filled with 3 L of three full-strength Hoagland nutrient solution (NS). The initial inoculum density was 96 g/m^2^. The full-strength Hoagland nutrient solution used had the following composition: 224 mg/L N, 235 mg/L K, 160 mg/L Ca, 62 mg/L P, 32 mg/L S, 24 mg/L Mg, 0.270 mg/L B, 1.12 mg/L Fe, 0.110 mg/L Mn, 0.131 mg/L Zn, 0.032 mg/L Cu, and 0.050 mg/L Mo. LM plants were exposed to artificial lighting conditions comprising 16 h of light and 8 h of darkness. The light period included 1 h of dawn, with a photosynthetic photon flux density (PPFD) of 125 µmol/s/m^2^, followed by 12 h of full light at a PPFD of 250 µmol/s/m^2^, with an RBG ratio of 36:37:36% and 1 h of dusk with a PPFD of 125 µmol/s/m^2^. The Daily Light Integral (DLI) was 11.70 mol/(m^2^·day). The temperature was maintained at 22 °C during the day and 20 °C at night, with a constant relative humidity of 65%. At harvest, each production unit (10 trays) was sampled. The biomass from five trays was pooled to generate one biological replicate, while the biomass from the remaining five trays was pooled to generate a second independent biological replicate (*n* = 2 biological replicates). The collected material was then freeze-dried using a PROLIO-4000 VHP Freeze Dry System (5pascal srl, Milano, Italy). The freeze-dried samples were subsequently ground to 500 μm with a laboratory mill (IKA A11, Staufen, Germany) to obtain a homogeneous powder.

### 2.2. Chemicals and Reagents

Trizma^®^ base, ≥99.9% (titration), urea BioUltra Molecular Biology, 99% (T), thiourea ReagentPlus^®^, ≥99.0%, dithiothreitol purity ≥99% (proteomics grade) (DTT) and iodoacetamide (proteomic grade), along with solvents for LC-HRMS/MS analysis, were purchased from Merck KGaA (Darmstadt, Germany) and VWR International PBI (Milan, Italy). Trypsin Gold Mass Spectrometry Grade was purchased from Promega (Milan, Italy). Disposable desalting cartridges PD-10 were purchased from Cytiva (Marlborough, MA, USA), while Sep-Pak C18 solid phase extraction (SPE) columns (1 cc, 50 mg) were obtained from Waters (Milford, MA, USA). HNO_3_ (Pure grade) was provided by Carlo Erba Reagents S.r.l. (Milan, Italy).

### 2.3. Mineral Profile of Lemna minor

For Al, K, Ca, P, Fe, Mg, Na, B, Cd, Co, Cr, Cu, Li, Mn, Mo, Pb, Zn determinations, freeze-dried samples were digested in a closed-vessel microwave digestion system (MARS 6, CEM Corporation, Matthews, NC, USA) with 10 mL of HNO_3_ (Pure grade, Carlo Erba) and analyzed by using inductively Coupled Plasma—Optical Emission Spectrometry (ICP-OES, Agilent Technologies, Santa Clara, CA, USA), as reported by D’ Imperio et al. [22]. To confirm the accuracy of the measurements of Nitrogen, NO_3_, Al, K, Ca, P, Fe, Mg, Na, B, Cd, Co, Cr, Cu, Mn, Mo, Li, Pb, Zn, certified reference vegetable material (CRM, NIST tomato leaf 1535a) was analyzed using the same procedure adopted for freeze-dried samples.

### 2.4. Total Protein Content Determination on Dry Weight

The total protein content (TPC) of freeze-dried LM samples was estimated according to the Dumas method on a CN 802 Carbon Nitrogen Elemental Analyzer provided by Velp Scientifica (VELP Scientifica Srl, located in Usmate, Italy). A total of 100 mg of freeze-dried LM sample was placed into a tin foil, purged with a carrier and dropped down into the combustion reactor to be converted into elements at a high temperature (>1000 °C). LM samples were analyzed in triplicate. Total nitrogen quantification was performed with an EDTA-based calibration curve and 5 concentration levels (range 0.5–10 mg). The number 6.25 was used as conversion factor from nitrogen to total protein and the results were expressed as % *w*/*w* [12]. Certified reference vegetable material (CRM, NIST tomato leaf 1535a) was analyzed to confirm the determination accuracy.

### 2.5. Protein Extraction and Purification Protocols

**Figure 1:**
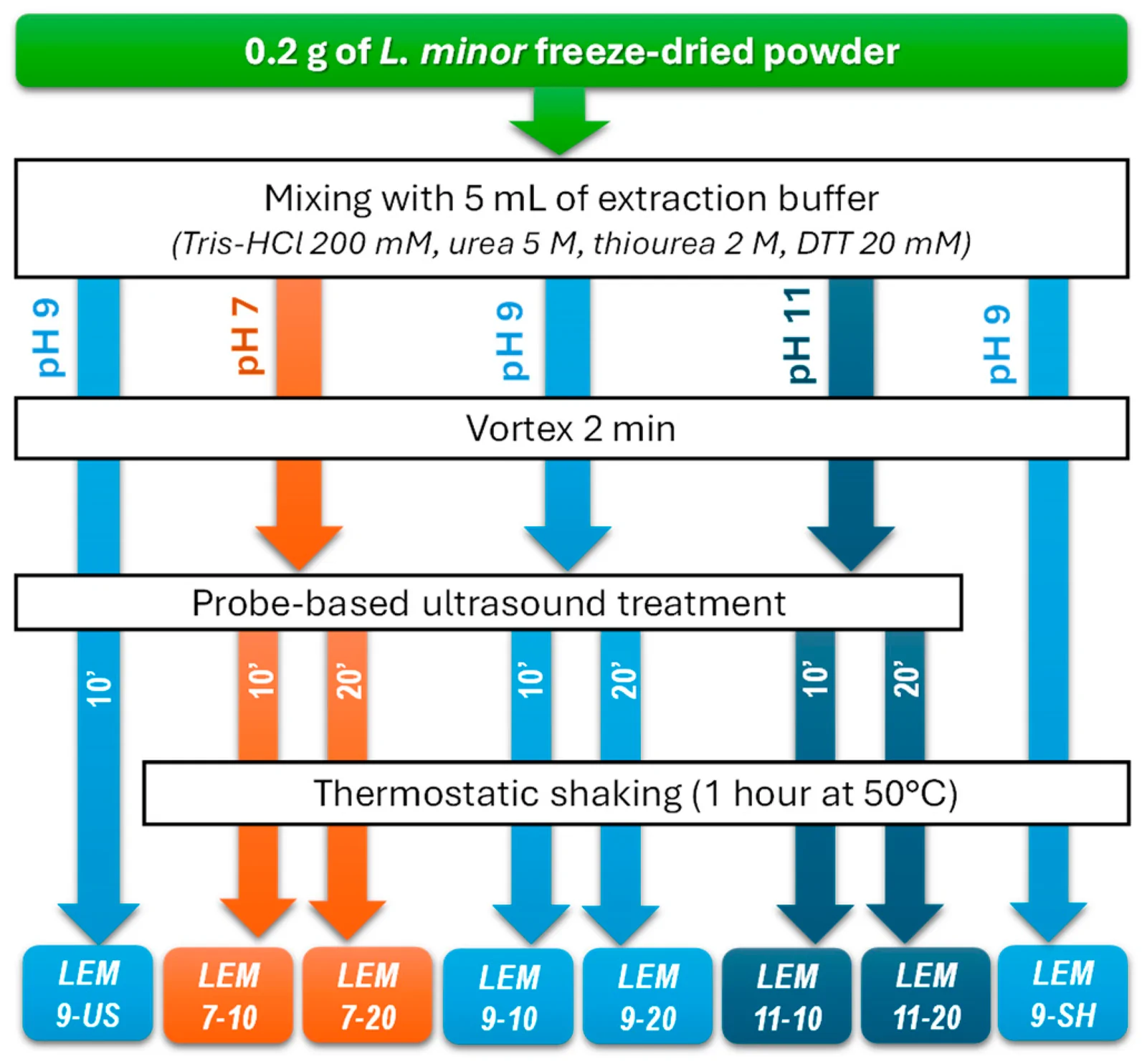
Scheme of the preparation protocols of duckweed protein extracts and relevant sample coding.

Two independent biological samples (*n* = 2) were used for the proteomic analysis and we submitted each to the different purification protocols tested. All the experiments were accomplished using a highly denaturant and reducing extraction buffer (Tris HCl 200 mM, Urea 5 M, Thiourea 2 M, DTT 20 mM). Four variables were considered to obtain a high protein extraction yield, namely (a) powder to solvent ratio (*w*/*v*), (b) buffer pH, (c) physical aids for cell disruption and (d) extraction time. Multiple extraction protocols were tested, including two different solid to solvent ratios (*w*/*v*) (1:10 and 1:25), three pH levels (7, 9 and 11), two physical aids (thermostatic incubation under orbital shaking and/or probe-based ultrasound treatment) and different exposure times to (10 and 20 min) probe-based sonication. Preliminary experiments revealed a gelation side-effect of the samples when the 1:10 solid to solvent ratio (*w*/*v*) was used; therefore, all the other tests were carried out at a fixed 1:25 ratio (*w*/*v*). As a general procedure, 0.2 g of LM lyophilized powder was mixed with 5 mL of extraction buffer. The samples were homogenized with a vortex for two minutes and then submitted to the steps depicted in Figure 1, according to the following conditions:

- Thermostatic incubation for 1 h at 50 °C under orbital shaking at 220 rpm (IKA, KS 4000 i control, IKA-Werke GmbH & Co. KG, Staufen, Germany);
- Ultrasound treatment with a probe sonicator (Vibracell^TM^, Sonics & Materials Inc., Newton, CT, USA) according to a pulsing program of 70% in amplitude, 10 s ON/5 s OFF pulse lengths, exposure time 10 or 20 min (sample overheating controlled with ice bath).

All the extracts were then centrifuged for 10 min at 4000× *g* (Sigma Laboratory Centrifuge 3K30, D37520 Osterode am Harz, Germany) and the supernatant filtered through 1.2 µm acetate cellulose (AC) membranes. Next, 2.5 mL of all samples were loaded on a disposable size exclusion column (G25 Sephadex column) and eluted with 3.5 mL of 50 mM ammonium bicarbonate to purify the protein extract, removing the low molecular weight interfering compounds including chlorophyll responsible for green pigmentation. Two technical replicates have been obtained for each sample procedure tested.

A ready-to-use colorimetric Bradford assay (Quick Start™ Bradford Protein Assay, Bio-Rad Laboratories s.r.l., Segrate, Italy) was used to estimate the total protein content of the resulting extracts and to calculate the extraction yield as the percental ratio to the TPC on dry weight assayed by Dumas. Samples were analyzed in duplicate and stored at −20 °C and filtered through 0.45 µm AC filters before analysis.

### 2.6. SDS-PAGE Analysis and In-Gel Tryptic Digestion

Protein extracts were profiled by means of sodium dodecyl sulphate–polyacrylamide gel electrophoresis (SDS-PAGE). Specifically, 20 µL corresponding to approximately 15 µg of proteins were separated under reducing condition on an 8–16% polyacrylamide pre-cast gel (13.3 cm × 8.7 cm × 1.0 mm) using Mini-Protean Tetra Cell equipment (Bio-rad Laboratories, Segrate, Italy) [23]. After running, gels were stained with Coomassie Brilliant Blue G-250 solution and the protein profiles detected on a ChemiDoc™Imaging System (Bio-Rad Laboratories, Segrate, Italy). Precision Plus Protein™ all blue standard (10–250 kDa, Bio-Rad Laboratories, Segrate, Italy) was used as a protein reference for molecular weight. Selected protein bands were manually excised from stained gels and subjected to in-gel digestion according to the protocol described by De Angelis et al. [24]. After drying, each sample was re-suspended in 100 µL of a solution of H_2_O/acetonitrile (ACN) 95/5 + 0.1% formic acid (*v*/*v*) and 20 µL were injected into LC/MS apparatus.

### 2.7. In Solution Digestion

A total of 600 μL aliquots of purified extracts were subjected to a 16 h tryptic digestion after thermal denaturation, chemical reduction, and alkylation according to the procedure described by Pilolli et al. [25]. The resulting samples were then centrifuged at 16,000× *g* for 10 min and purified by solid-phase extraction (SPE) on a Sep-Pak C18 SPE column (1 cc, 50 mg) [26]. The purified extract was finally analyzed by liquid chromatography/tandem high resolution mass spectrometry (LC-MS/MS) analysis.

### 2.8. LC-HRMS/MS Analysis

LC-MS/MS analyses were performed on an Vanquish UHPLC system coupled to a Orbitrap Exploris™ 240 Mass Spectrometer (Thermo Fisher Scientific, Waltham, MA, USA). For the peptide chromatographic separation, a reversed phase Aeris™ 3.6 μm PEPTIDE XB-C18 column, 2.1 × 150 mm (Phenomenex, Torrance, CA, USA) was used, at a flow rate of 200 μL/min and constant temperature of 25 °C (solvent A: water + 0.1% formic acid; solvent B: acetonitrile + 0.1% formic acid). An elution multistep gradient was applied: 0 to 40 min, solvent B increased from 10% to 40%; 40 min, solvent B step to 50% and then kept constant for 8 min; 48 min, solvent B increased to 90% and kept constant for 10 min; and 58 min, solvent B down to 10% and kept constant for 12 min for column conditioning. The injection volume was 10 μL, and each extract was injected in duplicate.

The source parameters (HESI) were set as follows: sheath gas flow rate 30, auxiliary gas flow rate 10, spray voltage 3.4 kV, ion transfer tube temperature 225 °C and S-lens RF level 70%. Untargeted HRMS/MS was performed by Full-MS/dd-MS2 acquisition mode (top 20), positive ionization. MS parameters were set up as follows. Full scan: microscan 1, resolution of 60 k, automatic gain control (AGC) target set to custom, normalized AGC target of 300%, maximum injection time auto and scan range 200–2000 *m*/*z*. MS/MS parameters were set up as follows: dd-MS2: microscan 1, resolution 15 k, AGC target Custom, normalized AGC target 200%, auto maximum injection time mode, isolation window 1.6 *m*/*z*, dynamic exclusion 20 s, peptide charge states from +1 to +4 were selected for fragmentation, and higher-energy collisional dissociation fragmentation with a normalized collision energy 30%.

Raw MS data were processed by Proteome Discoverer v. 3.0.0.757 (Thermo Fisher Scientific). The identification involved the Araceae database retrieved from Uniprot (Taxon ID 4454, 131742 entries, downloaded from https://www.uniprot.org/taxonomy/4454, accessed on 8 May 2025). The workflow parameters for sequence matching were: (i) precursor ion and fragments mass tolerance 10 ppm and 0.05 Da; (ii) enzyme: trypsin, ≤2 missed cleavages; and (iii) fixed modification: carbamidomethyl-cysteine; variable modifications: methionine oxidation. The false discovery rate was set at 1%, and only proteins identified by ≥2 peptides and with PSMs and Score Sequest HT ≥ 2 and 3, respectively, were retained. As for protein annotation, information directly retrieved from the UniProtKB platform and Gene Ontology (GO) were used.

### 2.9. Statistical Analysis

Protein content estimated by colorimetric assay in the different extraction procedures (*n* = 2 technical replicates for each purification protocol and *n* = 2 technical replicates for Bradford assay) were statistically compared with the analysis of variance (one-way ANOVA), 95% confidence level and a Tukey post hoc test. The label-free quantitation of the identified proteins was carried out via software based on summed abundances of unique and razor peptides. The intensities of precursor ions were used for automatic calculations, and the abundances were normalized over the total peptide amounts for best comparability among samples. Abundances were expressed as arbitrary units and were scientifically valuable for differential analysis but no direct conversion into absolute protein content could be carried out because the experimental design was not aimed at absolute protein quantitation. The calculated abundances were used for protocol comparisons by heatmaps and hierarchical cluster analysis (HCA) based on Euclidean distance and Ward’s linkage. An Enrichment Chart was created via Proteome Discoverer software tools, providing a list of gene ontology terms, pathways, or protein families that are represented by the most effective protocol.

Statistical evaluations were performed on the dataset obtained from the LC-MS/MS technical replicates (comprising two extraction duplicates for each biological replicate, each injected into duplicate, yielding eight samples per protocol).

### 2.10. In Silico Allergenicity Prediction

Full-length protein sequences, identified by MS, were evaluated in silico for their potential allergenicity using the AllerCatPro (v 2.0) “https://allercatpro.bii.a-star.edu.sg/ (accessed on 15 September 2025)” online platform [27]. Only food-relevant matches were considered for discussion. AllerCatPro matches with strong evidence of sequence similarity were further validated through a multi-step bioinformatic approach including: (a) epitope mapping for each identified allergen via the Immune Epitope Database (IEDB, “https://www.iedb.org/home_v3.php (accessed on 9 October 2025)”); (b) multiple sequence alignments (MSA) between potential allergenic proteins and reference allergens using Clustal Omega (UniProt platform, “https://www.uniprot.org/align (accessed on 9 October 2025)”); and (c) visual inspection of the alignments to confirm the conservation of experimentally validated epitopes within the query proteins. Regarding the IEDB search, the parameters were set as follows: linear peptide—substring for epitope and human as host and allergic for disease (default setting for assay and MHC restriction).

Semi-quantitative investigation of the putative allergenic proteins in LM was accomplished by collating experimental abundances of all protein accessions assigned to a single allergen through AllerCatPro analysis. Error bars represent the propagated standard deviation derived from the individual protein accessions within each allergen group.

## 3. Results and Discussion

*Lemna minor* (LM) was cultivated in a walk-in growth chamber with a 10-day production cycle. This duration, determined based on fertilization and environmental parameters (light and temperature), was chosen to prevent complete coverage of the substrate and the consequent stress from self-shading, which negatively affected yield and quality [28]. Therefore, under the experimental conditions tested, this interval represented the optimal harvest time to maximize biomass without compromising vitality and the nutritional profile. On the tenth day, the production units were harvested, yielding an average production of 1.00 ± 0.15 kg/m^2^. This value is consistent with both our previous production trials and the data reported in the literature [29]. The protein content of LM found in this study was 35% on a dry weight basis, corresponding to 1.7 g/100 g fresh weight, consistent with the values reported in Regulation [12].

In the regulation authorizing the use of *Lemna* as a functional food [12], concern is expressed about the potentially high Mn levels, which are generally higher than those found in common leafy vegetables. As indicated in the EFSA scientific opinion [20], reducing the Mn content in the nutrient solution is the most effective strategy to limit the accumulation of this potential risk factor. In our study, the detected levels—2.17 mg Mn/kg fresh weight—were lower than the threshold considered safe by EFSA, which is 6 mg/kg fresh weight. These findings highlight that, in soilless cultivation systems, nutrient solution management represents one of the most effective strategies for modulating the nutrient content such as the protein and mineral profile of a food crop, as also reported in our previous studies conducted on different horticultural species [30]. The mineral profile of LM is provided in Table S1 of the Supplementary Material. As noted by other authors, this profile may vary depending on environmental conditions and on the composition of the nutrient solution, and therefore on nutrient availability [29].

### 3.1. Effect of pH and Ultrasound-Assisted Extraction on Lemna minor Protein Recovery

Previous investigations from the literature proposed multiple protocols for extraction of LM proteins, mainly aiming at the production of a protein isolate with high nutritional and techno-functional properties for potential application in the food and feed industry [5,16,17,31,32]. Based on this knowledge, in this investigation a step forward was made to provide a comprehensive characterization of the proteome profiling of LM representative samples produced under controlled and reproducible growth conditions. Multiple extraction protocols were applied to collect information, modulating buffer pH, physical aids and sonication time.

**Figure 2:**
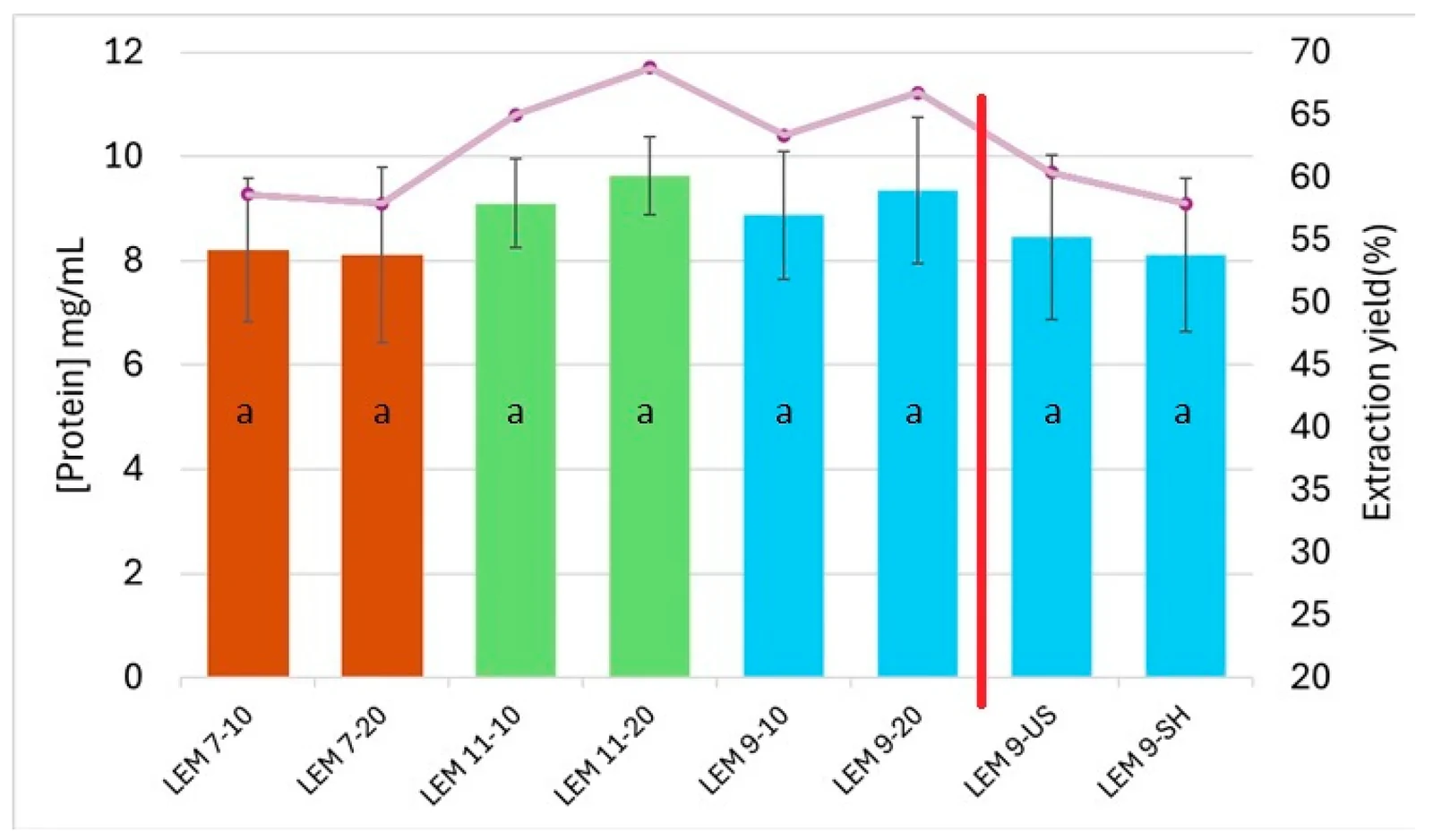
Protein content (bars) and extraction yield (line graph) of *L. minor* protein extracts obtained from duckweed powder using different extraction procedures described in Figure 1. The results of a Tukey–Kramer test for multiple mean comparisons are also reported and expressed using the following annotation: (a) no statistically significant difference found among samples.

As for buffer composition, highly denaturant and reducing conditions were selected as fixed points for the experimental plan. The effectiveness of this approach in plant proteome profiling was already reported by [33], providing high protein solubility. The different extraction protocols were initially compared based on total protein content and extraction yield according to a colorimetric assay of the protein extracts (Figure 2). Overall, no significant differences were observed among treatments, indicating that all procedures provided comparable results in terms of total protein recovery and extraction efficiency.

Specifically, extracts obtained at pH 7, 9, and 11 showed similar protein contents (8.2–9.1 mg/mL) and extraction yields (58.6–65.1%), suggesting that within the investigated range, pH had a limited effect on LM general protein extractability. These findings are consistent with previous studies reporting high protein solubility above neutral pH and a plateau effect under alkaline conditions [5,16,17,32].

Likewise, the comparison between conventional thermostatic extraction under shaking and ultrasound-assisted extraction revealed no significant differences in either protein content or extraction yield, with all treatments yielding approximately 8.5 mg/mL protein and ~60% recovery, probably due to the strong denaturing and reducing conditions, likely minimizing protein aggregation phenomena, and resulting in equivalent protein recoveries across all protocols.

Optimization of ultrasound duration further confirmed the robustness of the extraction procedure. Treatments of 10 or 20 min at 70% amplitude produced comparable protein contents and extraction yields irrespective of pH, with recoveries consistently around 60%. However, different extraction strategies may preferentially enrich specific proteins or protein classes according to their physicochemical properties, subcellular localization, or solubility. Therefore, despite the comparable extraction efficiencies observed, a comprehensive shotgun proteomics characterization of each extract was performed to assess the potential differences in protein composition and to identify complementary information that was capable of broadening the coverage of the *Lemna minor* proteome.

### 3.2. Shot-Gun Proteomics of Total Protein Extracts

#### Protein Identification

Protein extracts obtained using the different protocols were characterized by a bottom-up proteomics workflow, including tryptic digestion, untargeted high-resolution tandem mass spectrometry, and bioinformatic protein annotation. To obtain a comprehensive overview of the *Lemna minor* (LM) proteome, all datasets were combined and examined qualitatively, followed by comparative analyses to disclose protocol-dependent differences in protein class recovery.

One of the main challenges in proteomic research involving emerging plant species is the limited availability of sequence resources in public databases, due to the lack of complete and curated genomic and proteomic data. In the case of LM, only 121 protein sequences are currently available in the UniProtKB database (Taxonomy ID: 4472, last accession in October 2026), of which only 52 are fully reviewed. Similarly, only 244 Identical Protein Groups (IPG) are cataloged by NCBI under LM taxonomy, collating a high degree of sequence redundancy across total entries (38,820 accessions, last accession in October 2026). Clearly, these resources represent only a small fraction of the complex duckweed proteome. A well-established strategy to achieve comprehensive proteome coverage for species lacking dedicated databases is to query broader datasets from higher taxonomic levels, thereby exploiting sequence homology with the target organism [26]. In this investigation, a custom database encompassing the entire Araceae order was used (UniprotKB, Taxonomy ID: 4454), leveraging the high conservation of peptide sequences among phylogenetically close plant species. To address potential issues arising from the protein inference process when using a multi-species database, strict parsimony rules were applied during bioinformatic processing (e.g., grouping proteins sharing identical identified peptides). To ensure high identification confidence, a stringent filtering criterion requiring at least two unique peptides per protein was applied. This strategy enabled the identification of 1211 proteins and 4149 validated peptides after stringent filtering criteria, providing satisfactory coverage of the LM proteome (see Table S2). As expected, only a small fraction of proteins was directly assigned to LM species, whereas most identifications were associated with closely related ones, particularly *Spirodela intermedia*, *Colocasia esculenta*, and *Anthurium amnicola*. These homologous proteins from closely related duckweed species reflect either different gene isoforms or sequence variations between LM and the reference sequences of related *Araceae*. Consequently, while this approach avoids false collapses of distinct protein isoforms, it highlights that the 1211 identified proteins represent functional protein groups and sequence-resolved homologs rather than a strictly non-redundant gene count derived from a single-species genome.

**Figure 3:**
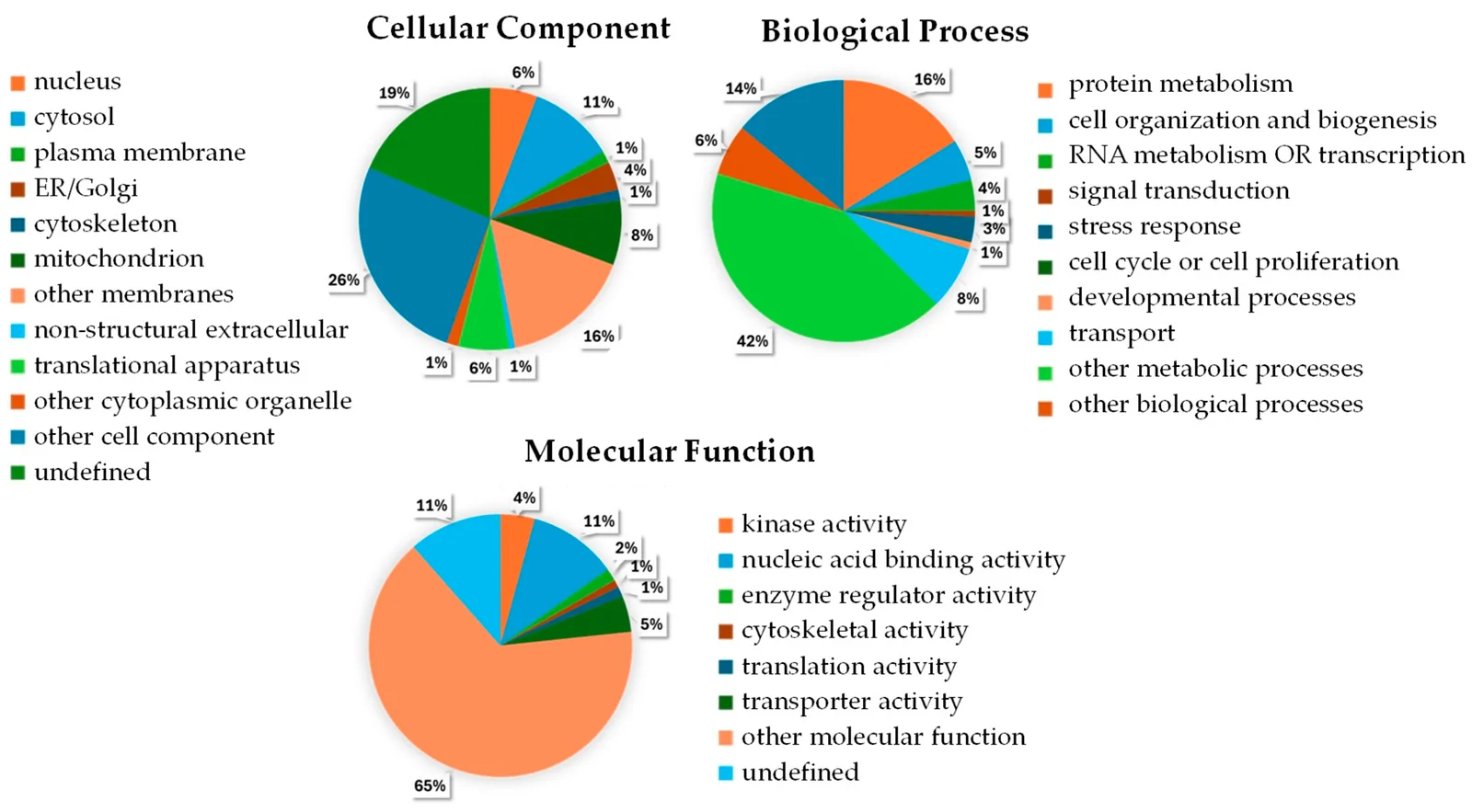
Graphical pie charts and color coding describing the cellular component, biological processes and molecular function of the shotgun-identified proteins.

Gene Ontology annotation revealed that the extracted proteins originated from multiple cellular compartments, including the nucleus, cytosol, mitochondria, and translational machinery, indicating that the extraction procedures provided a representative overview of the cellular proteome (see Figure 3).

Functionally, the identified proteins were predominantly associated with photosynthetic and primary metabolic processes (see Figure 3 and Table S2). Major protein classes included RuBisCo, ATP synthase subunits, photosystem I and II components, and chlorophyll a/b-binding proteins, all of which are essential for carbon fixation, light harvesting, electron transport, and energy metabolism [34,35,36,37]. Specifically, RuBisCo is a key enzyme of the Calvin cycle that mediates the assimilation of atmospheric carbon dioxide within photosynthetic tissues [34], while the subunits alpha and beta of ATP synthase are involved in regulation of ATP synthase activity [36]. Among the other proteins involved in photosynthetic pathways, the following were identified: Photosystem I P700 chlorophyll a apoprotein A1, which participates in electron transfer during the light phase of photosynthesis [35], Photosystem II CP47, an antenna protein associated with light-harvesting functions [38] and Chlorophyll a–b binding protein, which plays a critical role in capturing light energy and facilitating excitation transfer to the photosynthetic reaction centers [37].

Stress-response proteins, such as HSP70, were also detected, highlighting the presence of cellular defense mechanisms [26].

In addition, several enzymes involved in central carbon metabolism were identified, including glyceraldehyde 3-phosphate dehydrogenases, phosphoglycerate dehydrogenase, phosphoglycerate kinase, phosphoglycerate mutase, S-adenosylmethionine synthase, malate dehydrogenase and phosphoglycerate kinase. These last three enzymes were involved in methyl group transfer [39], in the citric acid cycle [40] and in glycolytic energy production [41], respectively. Structural proteins such as actins and tubulins associated with the cellular structure of the leaf were also identified along with numerous metabolic enzymes. Our results confirm the broad coverage of metabolic pathways revealed, further demonstrating the ability of the analytical approach to capture proteins involved in both cellular architecture and metabolic functions.

As previously mentioned, most of the literature concerning LM proteins has historically focused on optimizing extraction protocols, while a comprehensive characterization of this duckweed whole biomass proteome remains scarce. Other previous mass spectrometry studies on Lemnaceae proteome have been restricted to ecotoxicological stress responses (e.g., Loll et al. [18]) or comparative genomic/proteomic mapping in related species like *Spirodela polyrhiza* [19]. In Table 1, a comparison of the present study on LM whole-biomass proteomic and omics investigations in Lemnaceae is reported.

The high abundance of protein classes related to energy metabolism (such as RuBisCO, ATP synthase subunits, photosystem I and II components, and chlorophyll a/b-binding proteins) found in the LM proteome is in accordance with observations by Harkess et al., who reported these energy-related proteins to be overexpressed in *Spirodela* compared with *Arabidopsis thaliana*. This convergence suggests a strong biochemical similarity across duckweed species, which is further reinforced by our bioinformatic approach, where a significant portion of *L. minor* proteins showed high homology with *Spirodela* sequences.

The functional and nutritional significance of the identified LM proteome becomes particularly evident when compared to the traditional plant protein sources currently used in food systems, such as soybean (*Glycine max*) and yellow pea (*Pisum sativum*). Conventional legume protein concentrates are predominantly composed of seed storage globulins (e.g., glycinin, vicilin, and legumin), which are characteristically deficient in sulfur-containing amino acids (methionine and cysteine) and are frequently associated with major food allergenicity [42,43].

In contrast, our whole-biomass proteomic characterization highlights a high prevalence of photosynthetic and primary metabolic machinery, most notably RuBisCO and ATP synthase complexes. RuBisCO, representing a major fraction of the total identified protein pool, is widely recognized for its exceptionally well-balanced essential amino acid profile, which meets FAO/WHO nutritional guidelines and closely resembles reference animal proteins [44,45]. Furthermore, the presence of a diverse suite of metabolic enzymes (e.g., GAPDH, malate dehydrogenase) alongside structural proteins (actins and tubulins) provides a balanced metabolic matrix rich in essential amino acids [8], also contributing to favorable techno-functional properties, including high solubility and gelling capability [46].

**Figure 4:**
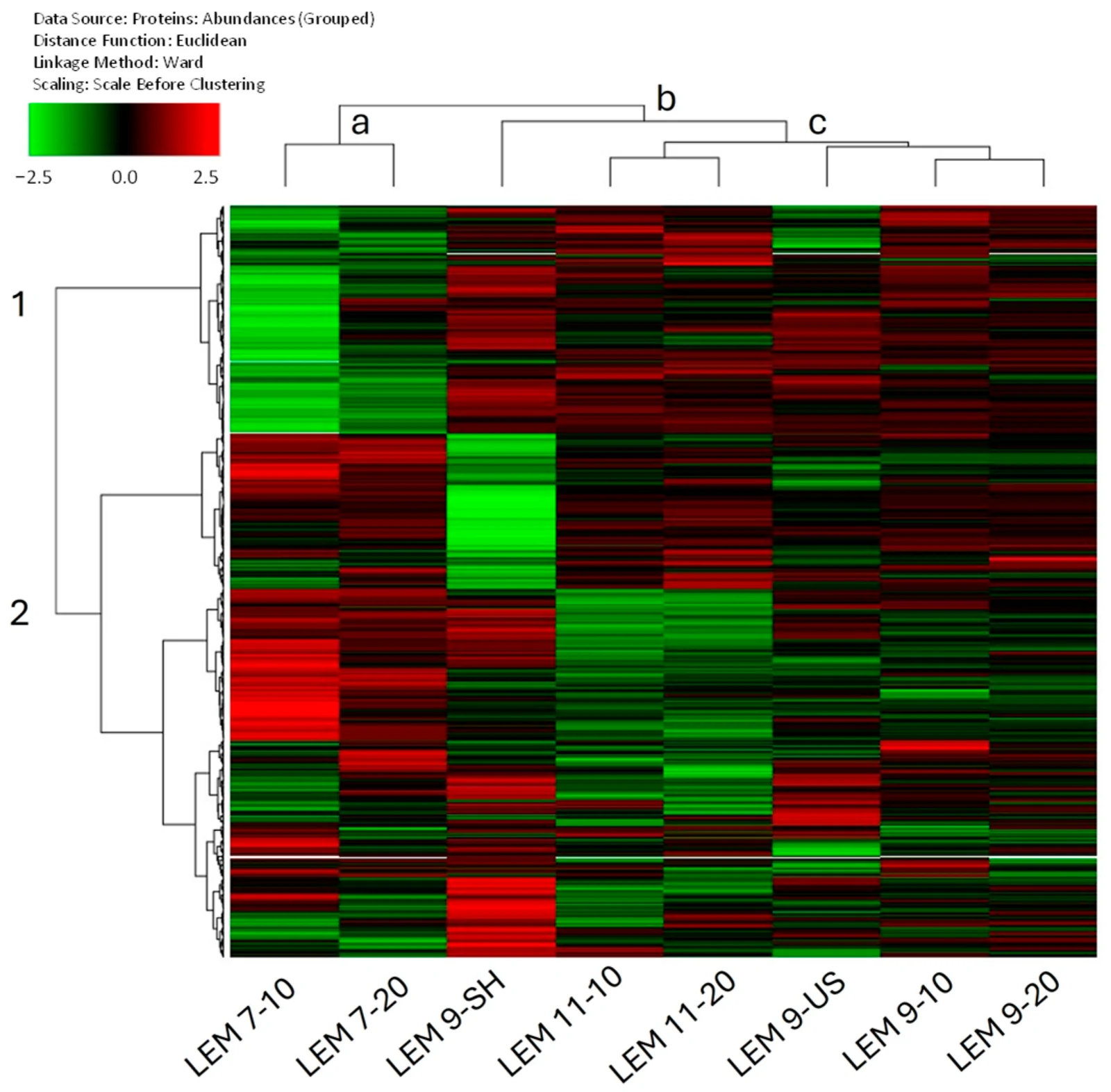
Hierarchical cluster analysis (HCA) and heat maps of protein accessions identified in the different extracts. HCA was carried out with the Euclidean distance function and the Ward linkage method for abundance values, which were scaled before clustering. Letters a, b and c mark clusters derived from sample similarity, while 1 and 2 refer to the main clusters produced by ion grouping.

Label-free quantification was performed on proteins identified against the Araceae database. Protein abundances were scaled and subjected to hierarchical cluster analysis (HCA), visualized as heatmaps with color-coded abundance distributions (Figure 4).

The resulting dendrograms revealed that samples clustered primarily according to extraction pH. Protocols performed at alkaline pH (9 and 11) showed highly similar protein profiles, whereas extracts obtained at pH 7 formed a separate cluster, indicating qualitative and quantitative differences in the extracted protein fractions. Gene Ontology enrichment analysis revealed that proteins favored by alkaline extraction were mainly associated with redox processes, primary metabolism, cofactor biosynthesis, and regulatory pathways (Tables S3 and S5), while neutral conditions preferentially enriched proteins involved in translation, nitrogen metabolism, ion homeostasis, and RNA-related functions (Table S4).

The contribution of the extraction aid was less pronounced. Samples sonicated for 10 or 20 min clustered closely together, suggesting that sonication time had only a limited impact on the overall proteome composition. LEM 9-10 and LEM 9-US displayed highly similar abundance profiles, whereas LEM 9-SH showed a partial shift toward the profile of LEM 11-10, suggesting a reduced extraction efficiency for specific protein subsets when shaking was used alone. Overall, these results indicate that ultrasound contributed to a broader recovery of proteins.

Although all protocols provided comparable total protein yields, they generated consistent differences in protein composition and abundance distributions, mainly at different pH values. These findings indicate that the extraction methods are not fully redundant and can selectively enrich distinct proteins or functional protein groups. Consequently, the information provided by the different extraction strategies should be regarded as complementary, contributing to a broader and more comprehensive characterization of the *Lemna minor* proteome than could be achieved using a single extraction protocol.

### 3.3. Electrophoretic Profile (SDS-Page)

The protein extracts obtained under the different experimental conditions were further characterized by SDS-PAGE, and representative electrophoretic profiles are shown in Figure S1A. Overall, the gels revealed qualitative differences among extraction protocols, particularly in the 10–50 kDa molecular weight (MW) range, highlighting the influence of extraction conditions on the recovery of specific protein fractions.

Extracts obtained under alkaline conditions (pH 9 and pH 11) exhibited highly comparable electrophoretic profiles regardless of sonication time. Prominent bands were consistently observed at approximately 14, 25, and 50 kDa, together with weaker bands distributed between 16 and 50 kDa. Among these samples, LEM 9-10 displayed the most intense band in the 50 kDa region, suggesting a more efficient recovery of proteins within this MW range. In contrast, extraction at pH 7 resulted in a markedly different profile characterized by a dominant 25 kDa band, weaker protein bands between 20 and 50 kDa, and a pronounced low-molecular-weight smear below 16 kDa, particularly in the LEM 7-10 sample. The presence of these smeared signals may indicate partial protein degradation or increased susceptibility of extracted proteins to denaturing conditions, likely promoted by the combined action of a neutral pH, strong chaotropic agents, and physical extraction treatments.

A similar trend was observed when comparing ultrasound-assisted extraction and thermostatic extraction under shaking. Samples LEM 9-US and LEM 9-SH displayed almost identical band patterns, closely resembling that of LEM 9-10, with well-defined bands at 14, 25, and 50 kDa. These results suggest that both ultrasound and mild heating promote comparable levels of cell disruption and protein solubilization, leading to the extraction of largely overlapping protein populations.

The electrophoretic observations were fully consistent with the hierarchical clustering analysis (HCA) performed on the shotgun proteomics dataset. In both approaches, samples extracted at pH 9 and pH 11 clustered closely together, indicating highly similar proteomic compositions, whereas extracts obtained at pH 7 formed a distinct profile. To further support the shotgun proteomics results and establish an electrophoretic map of the *Lemna minor* proteome resolved by SDS-PAGE, the most representative electrophoretic bands were excised and subjected to in-gel digestion followed by HRMS/MS analysis. To maximize protein recovery and assignment confidence, bands exhibiting comparable electrophoretic mobility across the different extraction protocols were pooled prior to analysis (Figure S1B). Protein identification was performed using a customized database generated from the proteins that were previously identified by shotgun proteomics, thereby increasing the reliability of band annotation. Proteins showing a coverage percentage ≥ 20% are summarized in Table 2, while the complete list of identified proteins is available in the Supplementary Material (Table S6).

Overall, the in-gel proteomic analysis confirmed the predominance of photosynthetic and primary metabolic proteins already highlighted by global proteomic investigation. The intense band at approximately 50 kDa (band A) displayed a particularly complex composition and was mainly associated with the large subunit of RuBisCO, in agreement with previous reports [5]. The same region also contained ATP synthase α- and β-subunits, Photosystem II CP47, elongation factor 1-α, Photosystem I P700 chlorophyll a apoprotein A1, and several metabolic enzymes. Similarly, the 37–50 kDa region (band B) was characterized by proteins involved in photosynthesis and energy metabolism, including ATP synthase subunits, RuBisCO activase, Photosystem II CP43, HSP70, S-adenosylmethionine synthase, malate dehydrogenase, and phosphoglycerate kinase.

The band migrating at approximately 30 kDa (band C) contained both photosynthetic and metabolic proteins, including cytochrome f, Photosystem II protein D1, 14-3-3-like proteins, and RuBisCO large-chain fragments.

The highly intense 25 kDa band (band D), particularly abundant in alkaline extracts, was mainly attributed to chlorophyll a/b-binding proteins, consistent with previous observations in duckweed proteomes [5]. This region also contained ATP synthase subunits, oxygen-evolving complex proteins, metabolic enzymes, and RuBisCO-derived peptides. Likewise, proteins migrating at approximately 20 kDa (band E) were predominantly associated with the photosynthetic apparatus, especially chlorophyll a/b-binding proteins and Photosystem II components.

Lower-molecular-weight bands also corresponded to biologically relevant proteins. The 16 kDa band (band F) was mainly assigned to the ATP synthase epsilon chain and glutaredoxin-dependent peroxiredoxin, whereas the 14 kDa band (band G) was dominated by the RuBisCO small subunit, together with histone H4. Notably, peptides derived from the RuBisCO large chain were detected in most electrophoretic bands. This observation suggests that the strongly denaturing extraction conditions may have promoted partial fragmentation of highly abundant proteins, generating polypeptides migrating at lower molecular weights. Consequently, the recurrent identification of the same protein in multiple bands is likely attributable to the presence of protein fragments and/or naturally occurring isoforms.

### 3.4. In Silico Allergenicity Risk Assessment of Lemna minor Using AllerCatPro

To assess the allergenic potential of *Lemna minor* (LM), the shotgun proteomics dataset was subjected to an in silico evaluation according to FAO/WHO and EFSA recommendations [21] using the AllerCatPro 2.0 platform. This tool predicts potential allergenicity based on sequence similarity, structural epitope conservation, and alignment with curated allergen databases. Proteins are classified as showing strong, weak, or no evidence of allergenicity, with an overall prediction accuracy of approximately 84% [27,47].

On the basis of the AllerCatPro search, among the 1211 proteins identified in the LM proteome, 40 protein accessions showed strong evidence of similarity to known allergens from fruits, vegetables, cereals, fish, and tree nuts. Twenty-five proteins met the criterion of >93% similarity in 3D epitope structure, whereas the remaining candidates were prioritized based on the FAO/WHO criterion of >35% sequence identity over an 80-amino-acid window. The curated list of LM proteins with putative allergenicity based on strong evidence is displayed in Table 3 together with all the information collected in terms of similarity with known allergenic proteins.

The strongest warning was observed for chlorophyll a/b-binding proteins, which showed high similarity to the celery allergen Api g 3 (see Table 3). This finding is particularly relevant because celery is one of the 14 allergens subject to mandatory labeling in the European Union and is associated with clinically significant allergic reactions, including anaphylaxis [48]. Although previous studies reported no direct cross-reactivity between LM and celery [20], the high structural similarity identified here suggests that additional in vitro and in vivo investigations are required, especially considering the growing interest in duckweed as a novel food source.

Other metabolic enzymes also exhibiting strong homology with known food allergens (see Table 3) were disclosed. These included glyceraldehyde-3-phosphate dehydrogenase, malate dehydrogenase, pectinesterase, chitinase, thaumatin-like proteins, fructose-bisphosphate aldolase, pyruvate kinase, and lactoylglutathione lyase, which showed similarity to allergens from mango, kiwi, banana, avocado, tomato, rice, maize, fish, and crustaceans. Notably, pectinesterase and an uncharacterized LM protein displayed similarity to the kiwi allergens Act d 7 and Act d 1, respectively, whereas a thaumatin-like protein showed high similarity to the banana allergen Mus a 4. Additional matches included an L-ascorbate peroxidase that was highly similar to the pepper allergen Cap a 30kD and an uncharacterized protein related to the tomato allergen Sola l 2. Although many of these source foods are not classified as priority allergens, fruit allergies are increasingly recognized worldwide and may cause reactions ranging from oral allergy syndrome to severe anaphylaxis [49]. More details on proteins with predicted allergenicity by the AllerCatPro 2.0 web server were reported in Table S7 of the Supplementary Material.

To give more insight into such in silico findings, allergen-specific epitopes were searched within the full-length sequences of proteins with putative allergenicity. Specifically, IEDB mapping and MSA were performed to identify experimentally validated linear B-cell epitopes and conserved motifs, providing specific details on shared epitope regions that could trigger cross-reactivity. Exact sequence conservation was observed between an LM protein and the validated epitope of the mango allergen Man i 1, while partial conservation was found for the rice allergen Ory s Glyoxalase I (see Figure S2A,B). Taking into account that computational sequence- and structure-based homology predictions reflect a potential risk of cross-reactivity rather than a confirmed, actual clinical allergenic hazard, the presence of conserved IgE-reactive epitopes further supports the need for dedicated in vivo allergenicity studies.

**Figure 5:**
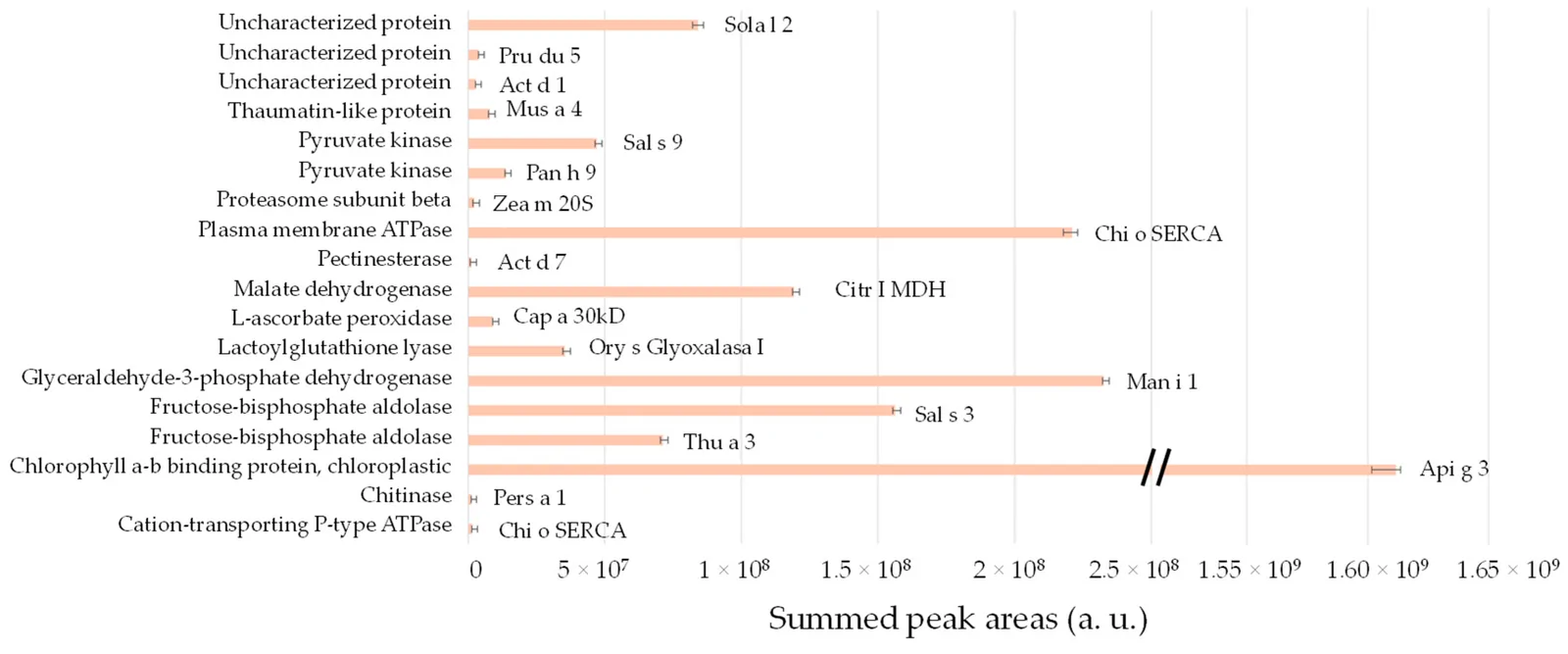
Semi-quantitative investigation of the abundance of potential allergenic proteins in the LEM 9-10 sample. Error bars represent the propagated standard deviation derived from the individual protein accessions (y-axis) within each allergen group. Protein abundance (x-axis) was determined by summing the peak areas of unique and razor peptides associated with a given protein accession.

Finally, a semi-quantitative proteomic analysis tailored to provide a preliminary overview of the distribution of potential allergens within the LM proteome has been carried out. Our results revealed that chlorophyll a/b-binding protein was by far the most abundant putative allergen in LM, reaching approximately 1.5 × 10^9^ a.u.: nearly one order of magnitude higher than any other candidate allergenic protein (see Figure 5). In contrast, proteins similar to Man i 1, Citr l MDH, Chi o SERCA, and Sal s 3 were detected at substantially lower abundances, while other predicted allergens were present only at trace levels. The combination of high abundance and strong similarity to the regulated allergen Api g 3 makes chlorophyll a/b-binding protein the primary allergenicity concern identified in this study. It is worth noting that peak area intensities do not directly correlate with IgE-binding affinity or clinical severity. However, identifying the most intense potentially cross-reactive protein signals provides a valuable starting point to prioritize candidates for future in vitro and in vivo clinical studies.

In conclusion, the in silico assessment suggests that LM may contain proteins with significant structural and sequence similarity to known food allergens. Although bioinformatic predictions cannot be considered conclusive evidence of clinical allergenicity, these findings serve to identify candidate LM proteins that warrant future in vitro (e.g., IgE-binding assays) and in vivo clinical validation before the large-scale introduction of LM-derived ingredients into the human diet [50].

## 4. Conclusions

This study provides the first comprehensive characterization of the *Lemna minor* proteome achieved through a high-resolution mass spectrometry-based shotgun proteomics approach. While LM is increasingly recognized as a promising novel food and sustainable protein source, detailed information on its protein composition has remained scarce because of limited genomic and proteomic resources. By combining multiple protein extractions, untargeted HRMS/MS analysis, bioinformatic annotation, electrophoretic protein mapping, and in silico allergenicity assessment, the present work establishes a robust reference framework for future investigations on this emerging crop.

Under controlled cultivation conditions, LM exhibited high productivity and a protein content consistent with values reported for authorized novel-food products. The extraction protocols investigated generated comparable total protein recoveries, indicating that pH, extraction aid, and sonication duration had limited effects on the overall extraction yield. Nevertheless, proteomic analyses demonstrated that these variables substantially influenced the recovery of specific protein subsets. The integration of datasets obtained from complementary extraction conditions proved essential for expanding proteome coverage and capturing proteins associated with distinct cellular compartments and biological functions.

Overall, 1211 proteins and 4149 validated peptides were identified, representing the most extensive LM proteomic dataset currently available. Functional annotation revealed a proteome that was largely dominated by chloroplast-associated proteins involved in photosynthesis, carbon fixation, light harvesting, and energy metabolism, including RuBisCO, ATP synthase subunits, photosystem components, and chlorophyll a/b-binding proteins. Numerous proteins involved in stress response, primary metabolism, and cellular structure were also detected. SDS-PAGE coupled with in-gel proteomics corroborated the shotgun results and enabled the assignment of the major electrophoretic bands composing the LM proteome.

Finally, the allergenicity screening highlighted several proteins with predicted similarity to known food allergens, identifying chlorophyll a/b-binding protein as the most abundant putative allergen. Although these findings require experimental validation, they emphasize the importance of including allergenicity assessments in the safety evaluation of duckweed-based foods.

Collectively, the proteomic dataset generated in this work represents a valuable foundation for advancing the nutritional, functional, technological, and safety characterization of *Lemna minor* and supporting its wider application as a sustainable food ingredient.

## Figures and Tables

**Table 1:** Comparison between previous omics investigations in Lemnaceae and the LM whole-biomass proteome investigated here.

Species	Analytical Platform	Database	Identified Proteins	Main Focus/Objective	Reference
*Lemna minor*	LC-HRMS/MS	*L. minor* reference genome + UniProt “duckweed” DB	Up-down regulated proteins after atorvastatin/bentazon exposure	Ecotoxicology/Screening of Modes of Action (MoA) for pesticides and drugs	[18]
*Spirodela polyrhiza*	LC-HRMS/MS	Predicted proteome DB from *Spirodela p*. + protein contaminants	1300 proteins shared with *A. thaliana +* 950 unique proteins	Genomic/proteomic profiling of *Spirodela polyrhiza* with respect to *A. thaliana*	[19]
*Lemna minor*	LC-HRMS/MS	*Araceae* DB	1211 proteins	Global constitutive proteome profiling of whole LM biomass	Present Study

**Table 2:** List of proteins identified by HPLC-MS/MS analysis followed by software data processing against the Araceae database of selected bands in-gel digested referred to different extracts of LM. Only proteins showing a % of coverage ≥ 20 were itemized in the table.

BAND	Accession	Description	Max Coverage [%]	# PSMs	# Unique Peptides	MW [kDa]	Score	# Peptides
A	G8D4C6, Q8WHH2, D6MYI7, A0A7S5SB76	Ribulose bisphosphate carboxylase large chain	39	151	3	49.6	428.58	20
	I1VWL9, A9L981	ATP synthase subunit alpha	28	14	8	55.1	30.78	9
	A0A7I8KWX4	ATP synthase subunit beta	33	26	13	59.3	77.24	14
	A0A843X963	Elongation factor 1-alpha	21	18	8	54.2	46.77	8
B	A0A7I8LI56	(S)-2-hydroxy-acid oxidase	25	28	4	40.2	57.30	8
	H6THB6	ATP synthase subunit beta	31	40	2	52.4	106.34	11
	A0A7I8IPI6, A0A7I8JF68, A0A7I8K457, A0A7I8L6N5	Glyceraldehyde-3-phosphate dehydrogenase	22	29	2	42.7	70.07	9
	A0A843TTK1, A0A1D1YFH7	Malate dehydrogenase	22	12	5	35.6	35.23	5
	A0A843V7X7, A0A7I8JGE8, A0A7I8JEK8	Phosphoglycerate kinase	20	10	2	41.0	23.75	5
	A0A7I8K6N1	Phoshoribulokinase	26	21	3	44.3	52.12	9
	J9QG68, Q8WHH2, D6MYI7, A0A7S5SB76	Ribulose bisphosphate carboxylase large chain	35	80	2	49.6	210.71	15
	A0A1D1YVF9	S-adenosylmethionine synthase	28	12	7	42.8	32.51	7
C	A0A1D1XG53	14-3-3-like protein	26	13	2	21.5	37.37	5
	D6MYI7, Q8WHH2, A0A7S5SB76	Ribulose bisphosphate carboxylase large chain	26	70	3	53.2	180.63	14
D	A9L981, I1VWL9	ATP synthase subunit alpha	25	25	10	55.0	69.83	10
	H6THB6, A0A7I8KWX4	ATP synthase subunit beta	33	34	3	52.4	86.68	13
	A0A7I8L983, A0A7I8JXC2, A0A7I8KG84, Q40185, A0A843WCK4	Chlorophyll a-b binding protein	40	31	6	31.6	90.51	8
	C7DRS8	Putative photosystem II oxygen evolving system 33 kDa subunit	32	4	2	16.2	10.17	3
	Q8WHH2, D6MYI7, A0A7S5SB76	Ribulose bisphosphate carboxylase large chain	26	64	2	49.6	166.29	11
E	A0A1D1ZAB4	Photosystem II protein	33	20	3	28.6	51.63	7
F	A9L9A2	ATP synthase epsilon chain	42	6	3	14.7	16.32	3
	Q4U4H9, A0A7S5SB76, F8SVU2, A0A843WAQ5	Ribulose bisphosphate carboxylase large chain	22	39	4	51.5	100.35	12
G	A0A843WZZ9	Histone H4	46	15	3	11.5	35.29	5
	F8SVU2, A0A7I8KWH2	Ribulose bisphosphate carboxylase small subunit	38	66	7	19.7	171.55	9

**Table 3:** List of 40 accessions identified among the 1211 query accessions, classified as having a predicted strong (S) allergenic potential based on similarity to known food protein allergens, as assessed using the AllerCatPro 2.0 online platform.

Description	Identified Accession(s) (Uniprot)	Predicted Most Similar Allergen (Non-Autoimmune Allergens)			Similarity Degree (% Identity)	
		Allergen	Species	Common Name	Linear 80 aa Window	3D Epitope
Glyceraldehyde-3-phosphate dehydrogenase	A0A7I8L4L2	Man i 1	*Mangifera indica*	mango	87.5–95.0	100.0
Malate dehydrogenase	A0A843TTK1; A0A7I8KHY2; A0A7I8JB56; A0A7I8KC41	Citr l MDH	*Citrullus lanatus*	watermelon	78.8–96.2	94.1–100.0
Uncharacterized protein	A0A7I8IH65	Act d 1	*Actinidia deliciosa*	Kiwi fruit	86.2	94.7
Pectinesterase	A0A7I8KWQ9	Act d 7	*Actinidia deliciosa*	Kiwi fruit	77.5	93.3
Thaumatin-like protein (Fragment)	A0A1D1YI01	Mus a 4	*Musa acuminata*	banana	91.2	94.7
Chitinase	A0A1D1XP70	Pers a 1	*Persea americana*	avocado	76.2	93.3
Chlorophyll a-b binding protein. chloroplastic	A0A7I8L983; A0A7I8KG84; A0A7I8KBI6; Q40185; A0A843WDI8; H6UDT8; A0A1D1ZLC3; A0A1D1ZLJ0; A0A7I8KGY4; A0A843WCK4; A0A7I8KUQ7	Api g 3	*Apium graveolens*	celery	45.6–98.8	-
Uncharacterized protein	A0A7I8LGB2	Pru du 5	*Prunus dulcis*	almond	83.8	-
Uncharacterized protein	A0A7I8K3G8	Sola l 2	*Solanum lycopersicum*	tomato	85.0	93.3
L-ascorbate peroxidase	A0A7I8KPD5	Cap a 30kD	*Capsicum annuum*	bell pepper	73.8	100.0
Lactoylglutathione lyase	A0A7I8KK42; A0A1D1ZGC6	Ory s Glyoxalase I	*Oryza sativa*	rice	41.2–82.5	-
Proteasome subunit beta	A0A7I8KPY2	Zea m 20S	*Zea mays*	maize	86.2	94.7
Fructose-bisphosphate aldolase	A0A843WWB7	Thu a 3	*Thunnus albacares*	tuna	80.0	94.1
	A0A7I8L791	Sal s 3	*Salmo salar*	salmon	77.5	93.3
Pyruvate kinase	A0A1D1XZ87; A0A7I8LFA2; A0A7I8L9G7; A0A7I8KHP7; A0A1D1YGS9	Sal s 9	*Salmo salar*	salmon	56.2–65.0	-
	A0A7I8KP62; A0A7I8K3T0; A0A7I8JCV7; A0A1D1Y7F3; A0A843UV07	Pan h 9	*Pangasianodon hypophthalmus*	iridescent shark catfish	55.0–75.0	-
Cation-transporting P-type ATPase N-terminal domain-containing protein	A0A843VBP5	Chi o SERCA	*Chionoecetes opilio*	crab	51.3	-
Plasma membrane ATPase	A0A7I8LH35	Chi o SERCA	*Chionoecetes opilio*	crab	42.5	-

## Data Availability

The original contributions presented in this study are included in the article/Supplementary Material. Further inquiries can be directed to the corresponding author.

## Supplementary Materials

The following supporting information can be downloaded at https://www.mdpi.com/article/10.3390/foods15213812/s1: Figure S1: (A) SDS-PAGE profiles of *Lemna minor* protein extracts obtained by different extraction strategies, (B) Scheme of the gel bands selected for LC-MS/MS analysis; Figure S2: (A) alignment of Uniprot accessions A0A7I8JF68 and A0A7I8L4L2 belonging to Glyceraldehyde-3-phosphate dehydrogenase protein with the allergen Man I 1 (ANQ43386.1). (B) alignment of Uniprot accessions A0A7I8KK42; A0A1D1ZGC6 belonging to Lactoylglutathione lyase with the allergen Ory s Glyoxalase I (Q948T6); Table S1: Mineral profile of *Lemna minor* L. cultivated in a walk-in growth chamber; Table S2: List of protein identified and quantified by shot-gun proteomic experiment and software based data processing (Proteome Discoverer 3.0, Thermo Fisher Scientific). Results of in-silico allergenicity prediction by AllerCatPro 2.0 web server were reported for each protein; Table S3: Gene Ontology (GO) enrichment between LEM 7-10 and LEM 9-10 (down-regulated); Table S4: Gene Ontology (GO) enrichment between LEM 7-10 and LEM 9-10 (up-regulated); Table S5: Gene Ontology (GO) enrichment between LEM 7-10 and LEM 11-10 (down-regulated); Table S6: List of electrophoretic bands identified after in-gel protein digestion and untargeted HR-MS/MS analysis; Table S7: List of protein with predicted allergenicity by AllerCatPro 2.0 web server were reported for each protein identified against Araceae database.
