# Supplementary material for: High-Resolution Mass Spectrometry-Based Proteomic Profiling of Lemna minor: Insights into Protein Composition, Extraction Strategies and Allergenicity Risk
Source: Foods. 2026 Oct 24;15(21):3812. doi: 10.3390/foods15213812 (PMC13762424; doi:10.3390/foods15213812)

Figure S1. Panel A: SDS-PAGE profiles of *Lemna minor* protein extracts obtained by different extraction strategies. Panel B: Scheme of the gel bands selected for LC-MS/MS analysis. Bands with similar molecular weights across the samples were combined to increase the sensitivity of the LC-MS/MS analysis. Each capital letter refers to a pool of bands analysed.

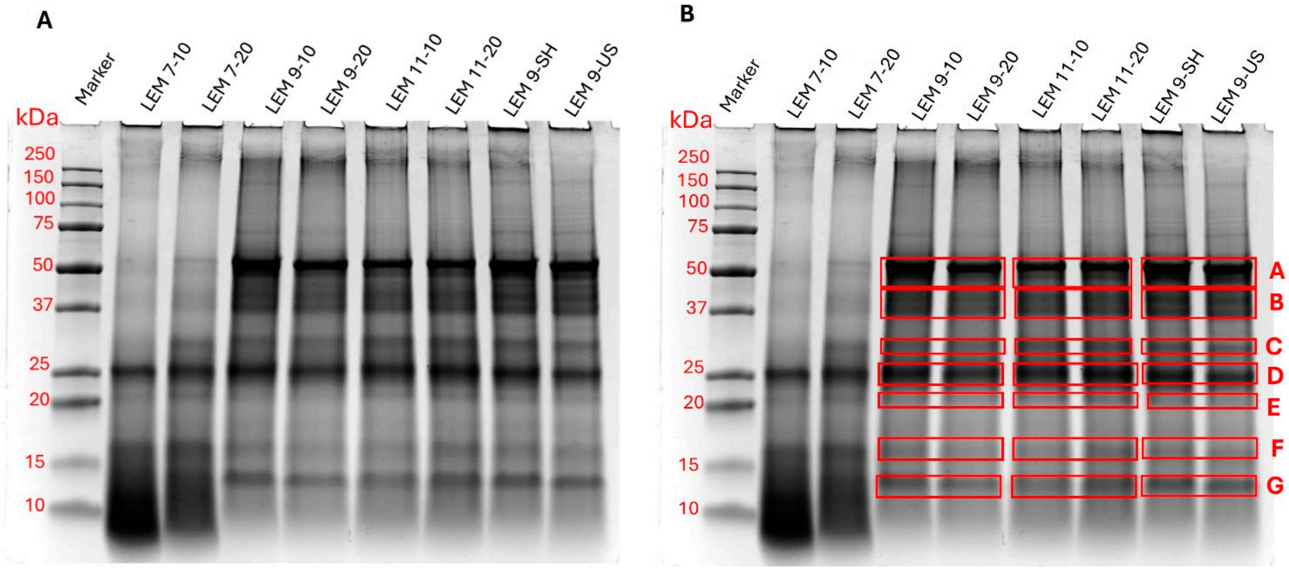

Supplement: Supplementary file 1 [file foods-15-03812-s001.zip › Figure S1.pdf]
