# Supplementary material for: High-Resolution Mass Spectrometry-Based Proteomic Profiling of Lemna minor: Insights into Protein Composition, Extraction Strategies and Allergenicity Risk
Source: Foods. 2026 Oct 24;15(21):3812. doi: 10.3390/foods15213812 (PMC13762424; doi:10.3390/foods15213812)

Figure S2. Panel A: alignment of Uniprot accessions A0A7I8JF68 and A0A7I8L4L2 belonging to Glyceraldehyde-3-phosphate dehydrogenase protein with the allergen Man I 1 (ANQ43386.1). Panel B: alignment of Uniprot accessions A0A7I8KK42; A0A1D1ZGC6 belonging to Lactoylglutathione lyase with the allergen Ory s Glyoxalase I (Q948T6). Alignments were performed by means UniProt Align tool (https://www.uniprot.org/align). Experimentally validated linear IgE epitopes are highlighted in the alignments in blue squares.

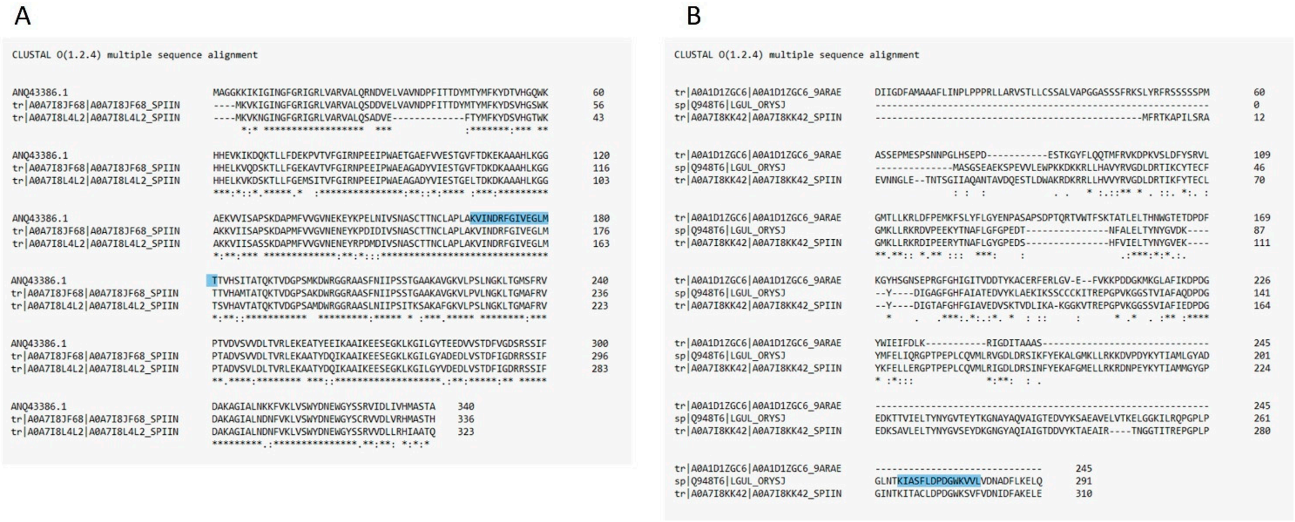

Supplement: Supplementary file 1 [file foods-15-03812-s001.zip › Figure S2.pdf]
